# Iron status and obesity-related traits: A two-sample bidirectional Mendelian randomization study

**DOI:** 10.3389/fendo.2023.985338

**Published:** 2023-02-14

**Authors:** Zengyuan Zhou, Hanyu Zhang, Ke Chen, Changqi Liu

**Affiliations:** ^1^ Department of Nutrition, Chengdu Women’s and Children’s Central Hospital, School of Medicine, University of Electronic Science and Technology of China, Chengdu, China; ^2^ Department of General Practice, Clinical Medical College & Affiliated Hospital of Chengdu University, Chengdu, China; ^3^ School of Exercise and Nutritional Sciences, San Diego State University, San Diego, CA, ;United States

**Keywords:** iron status, obesity-related traits, Mendelian randomization, serum ferritin, serum iron, transferrin saturation

## Abstract

**Background:**

The association between iron status and obesity-related traits is well established by observational studies, but the causality is uncertain. In this study, we performed a two-sample bidirectional Mendelian randomization analysis to investigate the causal link between iron status and obesity-related traits.

**Methods:**

The genetic instruments strongly associated with body mass index (BMI), waist-hip ratio (WHR), serum ferritin, serum iron, transferrin saturation (TSAT), and total iron-binding capacity (TIBC) were obtained through a series of screening processes from summary data of genome-wide association studies (GWAS) of European individuals. We used numerous MR analytical methods, such as inverse-variance weighting (IVW), MR-Egger, weighted median, and maximum likelihood to make the conclusions more robust and credible, and alternate methods, including the MR-Egger intercept test, Cochran’s Q test, and leave-one-out analysis to evaluate the horizontal pleiotropy and heterogeneities. In addition, the MR-PRESSO and RadialMR methods were utilized to identify and remove outliers, eventually achieving reduced heterogeneity and horizontal pleiotropy.

**Results:**

The results of IVW analysis indicated that genetically predicted BMI was associated with increased levels of serum ferritin (β: 0.077, 95% CI: 0.038, 0.116, P=1.18E-04) and decreased levels of serum iron (β: -0.066, 95% CI: -0.106, -0.026, P=0.001) and TSAT (β: -0.080, 95% CI: -0.124, -0.037, P=3.08E-04), but not associated with the levels of TIBC. However, the genetically predicted WHR was not associated with iron status. Genetically predicted iron status were not associated with BMI and WHR.

**Conclusions:**

In European individuals, BMI may be the causative factor of serum ferritin, serum iron, and TSAT, but the iron status does not cause changes in BMI or WHR.

## 1 Introduction

Obesity and iron deficiency (ID) are the most common nutritional diseases worldwide and have attracted great interest currently (1). Over the decades, the prevalence of obesity has substantially increased and reached pandemic levels (2), and ID anemia has become one of the leading causes of years lived with disability (3). The high prevalence of obesity and ID can lead to substantial health and economic burdens (4, 5).

As a nutritionally essential trace element, iron can affect the physical performance in hematopoiesis, transport oxygen, and various metabolic pathways (6). Given that, accumulating evidence has suggested the correlation between iron status and obesity since 1962 (7). Observational studies have found that obesity can predict lower iron absorption and affect iron metabolism, and body fat distribution (central obesity) is more likely to cause abnormal iron metabolism in women (8, 9). Additionally, previous studies suggested that inflammatory response characterized by obesity is involved in the regulation of iron metabolism (10, 11). A cross-sectional study found that serum ferritin was positively associated with lipid metabolism and abdominal obesity (12). Some studies have shown that iron homeostasis plays a crucial role in lipid accumulation and is a manifestation of obesity (13). Recently, studies indicated that the association between obesity and iron metabolism is reciprocal (14). Although observational evidence has recognized the association between obesity and iron status, it is difficult to assess the causal association.

The causal estimate of a modifiable phenotype or exposure to a disease is often of public health interest, but it is difficult or impractical to investigate the causality through randomized controlled trials (RCT). Mendelian randomization (MR) is an analytic approach using genetic variants as instrumental variables (IVs) for exposure, which can reduce the influence of confounders due to the premise that genotype only indirectly affects the disease state and is allocated during meiosis (15). It can provide a novel way to assess the causal association between potential factors and disease and solve existing reverse causation or confounding factors in observational epidemiology (16). To obtain causal estimates, MR analysis needs to fulfill the following three key assumptions: (1) genetic variants associated with the phenotype or exposure; (2) genetic variants have no association with confounding factors, and (3) genetic variants have no association with the disease or outcome except through the exposure (17). Thus, this method could provide new opportunities to elucidate the causal relationship between iron status and obesity-related traits.

In this study, we used body mass index (BMI) and waist and hip ratio (WHR) as obesity-related traits and four iron-related biomarkers for clinical evaluation of iron status to explore the causal relationship between iron status and obesity-related traits in European individual. We aimed to use the bidirectional MR analysis to supplement the findings of observational studies on whether iron status has an effect on obesity and vice versa.

## 2 Materials and methods

### 2.1 Study design

**Figure 1:**
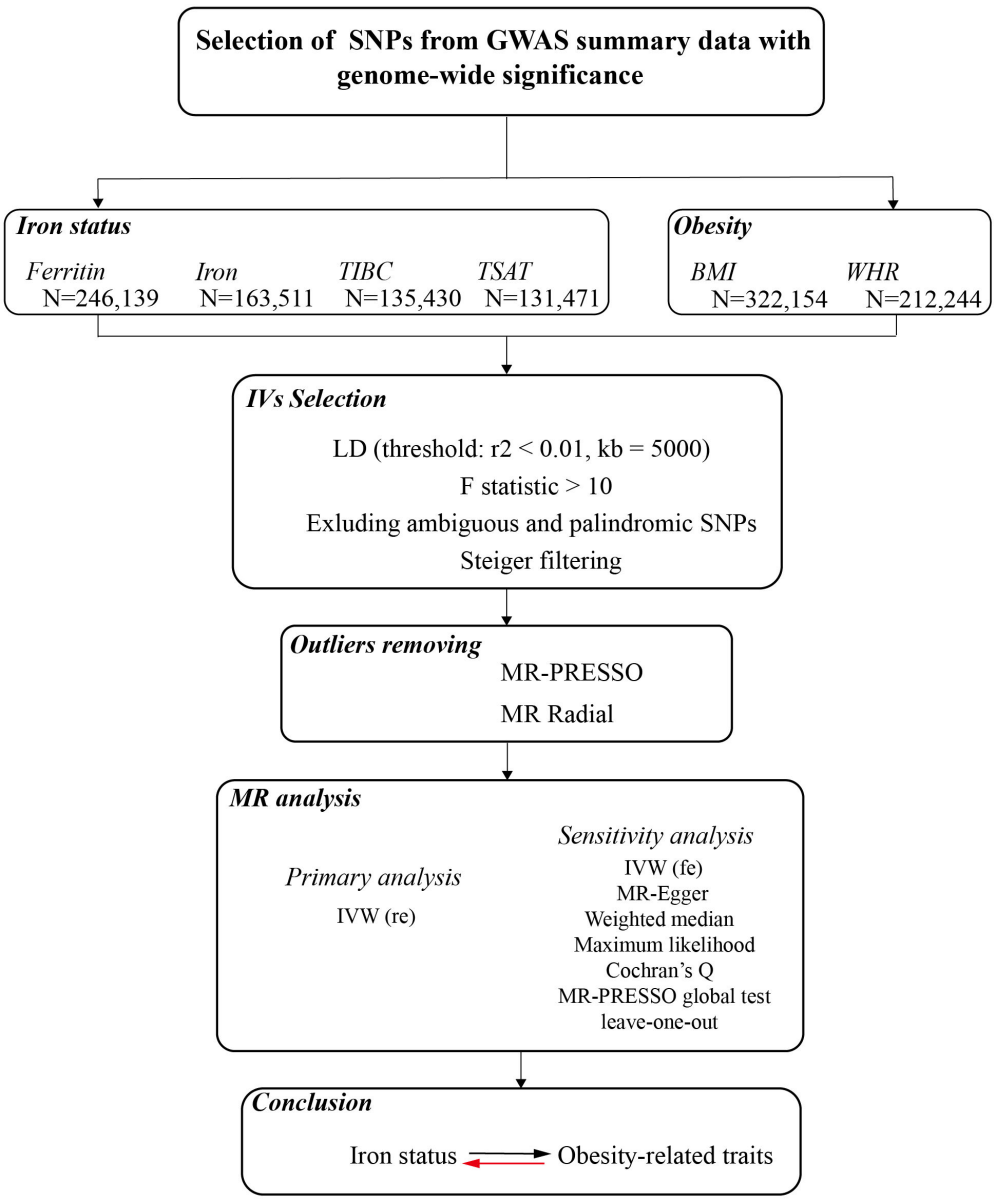
A flow diagram of the process in the current Mendelian randomization analysis.

This study described the bidirectional causality assessment of the relationships between iron status and obesity-related traits. Genetic instruments are required for both iron status-related biomarkers and obesity-related traits, and MR analysis was performed in both directions. The data of the instrumental variable are publicly available from the summary statistics among European populations, and no ethical approval was required. The overall design of our study is shown in **Figure 1**.

### 2.2 Data sources and instruments

#### 2.2.1 Iron status

We obtained the genetic instruments based on the latest genome-wide association study (GWAS) data on iron status, which performed a meta-analysis combining GWAS results from Iceland (deCODE genetics), United Kingdom (INTERVAL study) and Denmark (Danish Blood Donor Study) for four iron-related biomarkers: serum ferritin (up to N=246,139 individuals), serum iron (up to N=163,511 individuals), TIBC (up to N=135,430 individuals), and TSAT (up to N=131,471 individuals) (18). For the iron status, serum ferritin reflects stored iron, serum iron reflects circulating iron, and transferrin saturation reflects iron availability, which is derived as serum iron divided by the total iron-binding capacity (TIBC) from Genetics of Iron Status (GIS), and the TIBC as measured directly.

#### 2.2.2 Obesity-related traits

Genetic instruments with obesity-related traits were obtained from the Genetic Investigation of Anthropometric Traits (GIANT) Consortium. The BMI data were derived from a two-stage meta-analysis of 322,154 individuals of European descent (19), and WHR data were obtained from a meta-analysis of GWAS in 212,244 European individuals (20). **Supplementary Table 1** summarizes the data sources used in this study.

Genetic variants were extracted from each published GWAS at the genome-wide significance level (P<5×10^−8^) for each exposure. To ensure that each single nucleotide polymorphism (SNP) was independent of the other, we assessed the linkage disequilibrium (LD) between the SNPs, satisfying the criteria of r^2^ = 0.001 and kb=5,000. If an exposure-associated index SNP was absent from the outcome data set, a proxy SNP was not used. In addition, we performed variant harmonization of the genetic variants by combining two or more independently generated data. It is necessary to ensure that genetic variants from all publicly available datasets are consistent and allele mismatches are avoided, which can lead to bias in the causal effect estimate (21). Palindromic and ambiguous SNPs were removed from the analysis. The R^2^ and F values for each trait were calculated based on the derived summary statistics (**Supplementary Tables 2-7**). The F statistic can reflect the strength of instrumental variants (IVs), and a threshold of F < 10 has been used to define a weak IV, which is well-accepted in the field. Thus, we screened the F statistic of IVs > 10 in our analysis, ensuring that the relative bias in effect estimations caused by weak IVs was < 10% (22). This was calculated using the following formula:


$$F=\;\left(R^{2}/k\right)/\left(\left[1-R^{2}]/[n-k-1\right]\right)$$

R^2^, which is as an indicator of power for MR studies, is defined as the proportion of variability in the exposure explained by genetic variants; k is the number of instruments used in the model; and n is the sample size.

### 2.3 Mendelian randomization

In this study, we consider several methods, including Inverse variance weighted (IVW), MR-Egger regression, weighted median, and maximum likelihood, to weigh the estimated impact between exposure and outcome (23). The IVW was used as the most common method to assess the MR estimates for the causal effect under the assumption of balanced pleiotropy (24). The multiplicative random-effects model IVW was used as the primary analysis to avoid heterogeneity bias (25). Meanwhile, other MR methods have also been used to provide more accurate estimates, one of which is the MR-Egger regression, which differs from the IVW method by allowing a non-zero intercept. Additionally, the intercept term can be used to predict the directional pleiotropic effects (26). The weighted median estimator method was consistent even if 50% of the genetic variants were invalid (27). The maximum likelihood method estimates the probability distribution parameters by maximizing the likelihood function, and the bias is small (28). Indeed, these methods have relatively low statistical power compared to the IVW method, which was mainly designed to confirm consistent effects estimates seen in the main IVW estimate to determine the reliability of the results.

### 2.4 Sensitivity analysis

After the causal effect was detected using the above methods, we performed sensitivity analyses to assess the robustness of these findings to the assumption of balanced pleiotropy, including heterogeneity and the pleiotropy effect. Cochran’s Q was used in our study to identify heterogeneity in MR analysis (29). Additionally, we focused on methods that can test the bias from the horizontal pleiotropic effect. The MR-Egger intercept represents the average pleiotropic effect across genetic variants. If the intercept differs from zero (MR-Egger test), there is directional pleiotropy evidence (30). Then, the MR pleiotropy residual sum and outlier (MR-PRESSO) test removed the variant in question and refused IVW regression to identify horizontal pleiotropic outliers in the MR context (31). To improve the visualization of the IVW, we performed radial variants of the IVW instead of a scatter plot. The RadialMR imaging was used to complete the automated detection of outliers (32). The leave-one-out analysis was performed to assess whether the potentially pleiotropic SNPs affected causal estimates. In addition, we used MR Steiger to infer the direction of causality in our hypothesis. The threshold of the P-value was < 0.05, which is likely to be correct about the causal direction (33).

### 2.5 Statistical analyses

The TwoSampleMR package (34) and the RadialMR package (32) were used to perform the analysis in R (version 4.0.3). A P < 0.05 was defined as a threshold for statistical significance. The Bonferroni correction redefined the threshold for statistical significance in multiple testing (P < 0.05/n), where n refers to the number of MR tests (35). The adjusted P-values were 0.025 (0.05/2) for the forward MR analysis and 0.0125 (0.05/4) for the reverse MR analysis.

## 3 Results

### 3.1 Iron status and obesity-related traits

**Figure 2:**
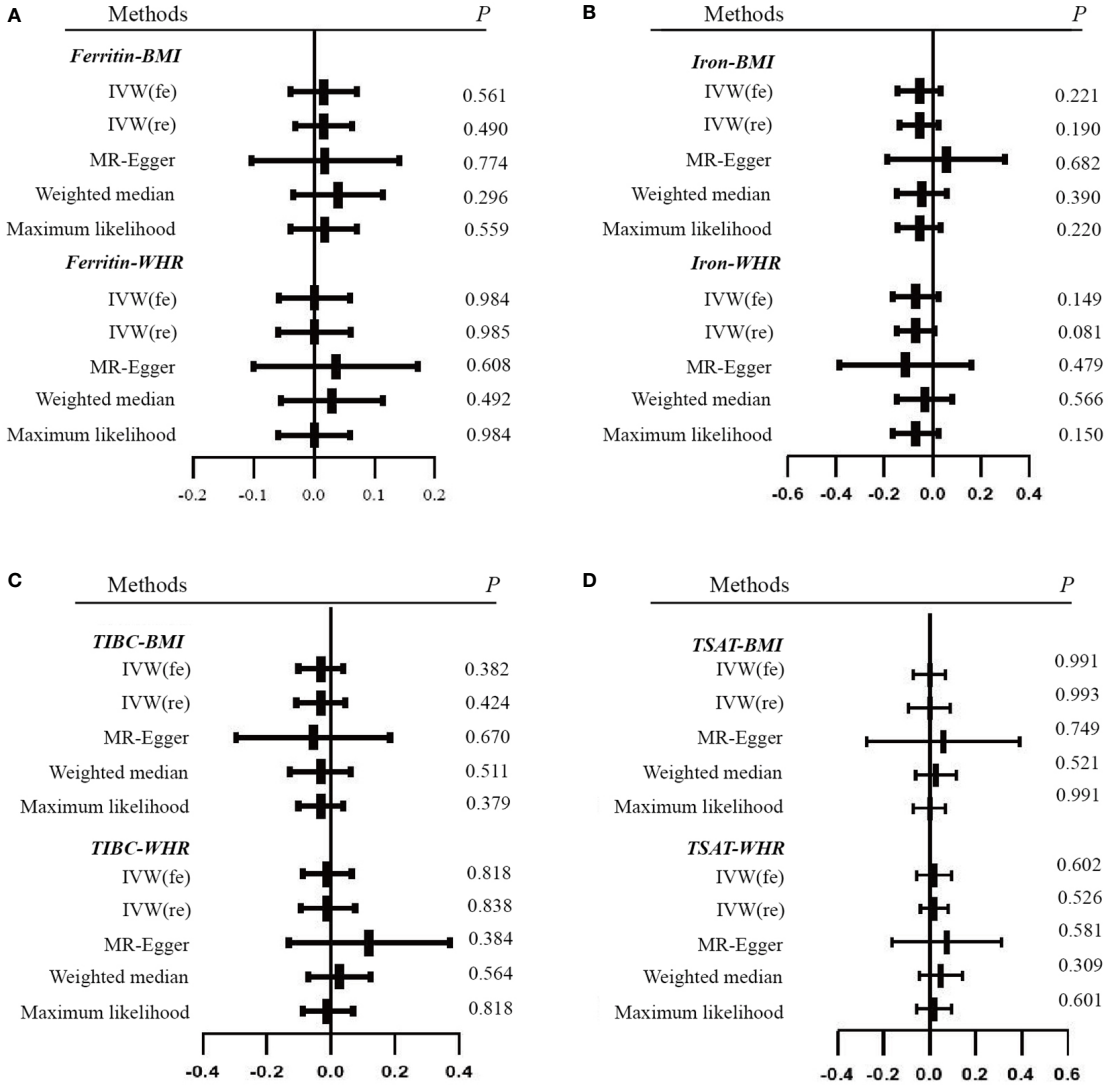
Forest plots of Mendelian randomization analyses of the association between genetically predicted iron status and obesity-related traits. **(A)** serum ferritin – BMI and WHR; **(B)** serum iron – BMI and WHR; **(C)** TIBC – BMI and WHR; **(D)** TSAT - BMI and WHR. Data are expressed as raw β with 95% CI. IVW, inverse variance–weighted method; fe, fixed effects model; re, multiplicative random effects model; BMI, body mass index; WHR, waist-hip ratio; TIBC, total iron-binding capacity; TSAT, transferrin saturation.

**Figure 3:**
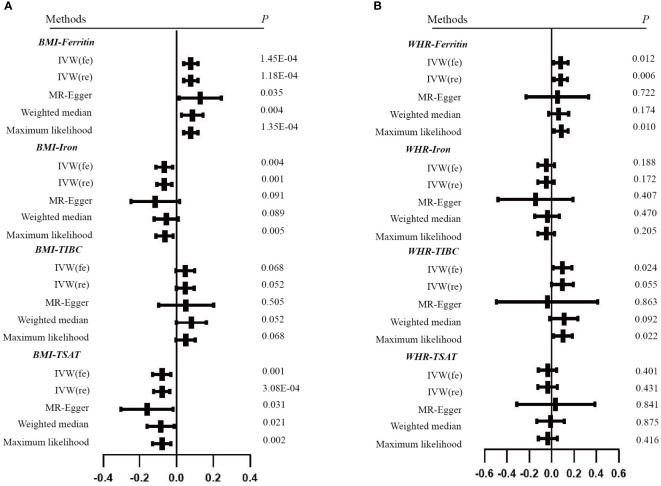
Forest plots of Mendelian randomization analyses of the association between genetically predicted iron status and obesity-related traits. **(A)** BMI - serum ferritin, serum iron, TIBC and TSAT; **(B)** WHR - serum ferritin, serum iron, TIBC and TSAT. Data are expressed as raw β with 95% CI.

In this study, the four biomarkers of iron status constituted the exposure and obesity-related traits were set as the outcomes. A total of 23 SNPs of serum ferritin, 6 SNPs of serum iron, 8 SNPs of TIBC, and 6 SNPs of TSAT remained (**Supplementary Tables 2-7**) after filtering and variant harmonization. The F statistics for the four biomarkers of iron status ranged from 10 to 86. The detailed MR estimates from different methods of assessing the causal effect are available in **Supplementary Tables 8-13**, which are presented as forest plots (**Figures 2**, **3**).

With the IVW method as the primary analysis, there was no association between the genetically predicted iron status biomarkers and the risk of obesity, including serum ferritin for BMI (β = 0.016, 95% CI: -0.030, 0.063, P = 0.490) and WHR (β = 0.001, 95% CI: -0.060, 0.061, P = 0.985); serum iron for BMI (β = -0.055, 95% CI: -0.137, 0.027, P = 0.190) and WHR (β = -0.070, 95% CI: -0.148, 0.009, P = 0.081); TIBC for BMI (β = -0.031, 95% CI: -0.107, 0.045, P = 0.424) and WHR (β = -0.009, 95% CI: -0.094, 0.077, P = 0.838); TSAT for BMI (β = 0, 95% CI: -0.091, 0.091, P = 0.993) and WHR (β = 0.020, 95% CI: -0.041, 0.081, P = 0.526). The MR Egger, weighted median, and maximum likelihood had the same effects as the IVW estimates. Moreover, the sensitivity analyses suggested no evidence of pleiotropy or heterogeneity (**Table 1** and **Supplementary Tables 8-11**). The MR-PRESSO and RadialMR outliers were removed to obtain the robust results (**Supplementary Tables 2-5** and **Supplementary Figure 1**). The result of the leave-one-out plot indicated that excluding any single SNP of the genetic variants hardly biased the outcome (**Supplementary Figure 3**). The results of the MR Steiger directionality test illustrated the accuracy of our estimate of causal direction (**Supplemental Tables 2-5**).

**Table 1:** MR results of the causal estimates of genetically predicted iron status on obesity-related traits.

Outcome Exposure		BMI			WHR		
	method	SNPs	β (95% CI)	P	SNPs	β (95% CI)	P
**Ferritin**	IVW (fe)	19	0.016(-0.039, 0.072)	0.561	20	0.001(-0.058, 0.060)	0.984
	IVW (re)	19	0.016(-0.030, 0.063)	0.490	20	0.001(-0.060, 0.061)	0.985
	MR Egger	19	0.018(-0.103, 0.140)	0.774	20	0.036(-0.100, 0.172)	0.608
	Weighted median	19	0.039(-0.034, 0.113)	0.296	20	0.03(-0.055, 0.114)	0.492
	Maximum likelihood	19	0.017(-0.039, 0.072)	0.559	20	0.001(-0.059, 0.060)	0.984
**Iron**	IVW (fe)	5	-0.055(-0.143, 0.033)	0.221	5	-0.07(-0.164, 0.025)	0.149
	IVW (re)	5	-0.055(-0.137, 0.027)	0.190	5	-0.07(-0.148, 0.009)	0.081
	MR Egger	5	0.056(-0.187, 0.299)	0.682	5	-0.112(-0.385, 0.160)	0.479
	Weighted median	5	-0.045(-0.148, 0.058)	0.390	5	-0.034(-0.149, 0.082)	0.566
	Maximum likelihood	5	-0.055(-0.144, 0.033)	0.220	5	-0.07(-0.165, 0.025)	0.150
**TIBC**	IVW (fe)	8	-0.031(-0.100, 0.038)	0.382	8	-0.009(-0.085, 0.067)	0.818
	IVW (re)	8	-0.031(-0.107, 0.045)	0.424	8	-0.009(-0.094, 0.077)	0.838
	MR Egger	8	-0.055(-0.295, 0.185)	0.670	8	0.12(-0.131, 0.371)	0.384
	Weighted median	8	-0.032(-0.126, 0.063)	0.511	8	0.028(-0.068, 0.124)	0.564
	Maximum likelihood	8	-0.031(-0.101, 0.039)	0.379	8	-0.009(-0.086, 0.068)	0.818
**TSAT**	IVW (fe)	5	0(-0.069, 0.070)	0.991	5	0.02(-0.055, 0.094)	0.602
	IVW (re)	5	0(-0.091, 0.091)	0.993	5	0.02(-0.041, 0.081)	0.526
	MR Egger	5	0.059(-0.271, 0.389)	0.749	5	0.075(-0.164, 0.315)	0.581
	Weighted median	5	0.029(-0.060, 0.118)	0.521	5	0.048(-0.044, 0.139)	0.309
	Maximum likelihood	5	0(-0.070, 0.071)	0.991	5	0.02(-0.055, 0.094)	0.601

IVW, inverse variance–weighted method; fe, fixed-effects model; re, multiplicative random-effects model; BMI, body mass index; WHR, waist-hip ratio; TIBC, total iron-binding capacity; TSAT, transferrin saturation; SNP, Single-nucleotide polymorphism. The bold font means P < adjusted p-values (0.025).

### 3.2 Obesity-related traits and iron status

For the reverse MR, the obesity-related traits constituted the exposure and the biomarkers of iron status were the outcomes. We screened 58 and 24 SNPs associated with BMI and WHR (**Supplementary Tables 6-7**). The F statistics for the obesity-related traits ranged from 10 to 97. The primary analysis revealed that obesity-related traits and iron status had a significant causal association after excluding the outlier SNPs. Specifically, the BMI for serum ferritin is β = 0.077, 95% CI: 0.038,0.116, P = 1.18E-04; for serum iron β = -0.066, 95% CI: -0.106, -0.026, P = 0.001 and for TSAT β = -0.080, 95% CI: -0.124, -0.037, P = 3.08E-04. However, we found that genetically predicted BMI was not associated with TIBC. The robustness of these results has been validated with additional methods (**Table 2** and **Supplementary Table 12**). Cochran’s Q and MR Egger intercept tests showed no evidence of instrumental heterogeneity and horizontal pleiotropy for serum ferritin, serum iron and TSAT (P > 0.05). Leave-one-out analysis indicated that the causality was not driven by any SNP (**Supplemental Figure 4**).

**Table 2:** MR results of the causal estimates of genetically predicted obesity-related traits on iron status.

Outcome Exposure		Ferritin			Iron			TIBC			TSAT		
	method	SNPs	β (95% CI)	P	SNPs	β (95% CI)	P	SNPs	β (95% CI)	P	SNPs	β (95% CI)	P
**BMI**	IVW (fe)	48	0.077 (0.037, 0.117)	**1.46E-04**	48	-0.066 (-0.112, -0.021)	**0.004**	53	0.048 (-0.004, 0.100)	0.068	55	-0.08 (-0.129, -0.031)	**0.001**
	IVW (re)	48	0.077 (0.038, 0.116)	**1.18E-04**	48	-0.066 (-0.106, -0.026)	**0.001**	53	0.048 (0, 0.096)	0.052	55	-0.08 (-0.124, -0.037)	**3.08E-04**
	MR Egger	48	0.128 (0.013, 0.242)	0.035	48	-0.116 (-0.248, 0.016)	0.091	53	0.051 (-0.098, 0.201)	0.505	55	-0.161 (-0.303, -0.019)	0.031
	Weighted median	48	0.086 (0.027, 0.144)	**0.004**	48	-0.056 (-0.122, 0.009)	0.089	53	0.080 (-0.001, 0.161)	0.052	55	-0.086 (-0.160, -0.013)	**0.021**
	Maximum likelihood	48	0.078 (0.038, 0.118)	**1.35E-04**	48	-0.065 (-0.111, -0.020)	**0.005**	53	0.049 (-0.004, 0.101)	0.068	55	-0.08 (-0.129, -0.030)	**0.002**
**WHR**	IVW (fe)	20	0.082(0.018, 0.146)	**0.012**	20	-0.05(-0.125, 0.025)	0.188	21	0.097(0.013, 0.182)	**0.024**	18	-0.035(-0.118, 0.047)	0.401
	IVW (re)	20	0.082(0.023, 0.141)	**0.006**	20	-0.05(-0.122, 0.022)	0.172	21	0.097(-0.002, 0.196)	0.055	18	-0.035(-0.124, 0.053)	0.431
	MR Egger	20	0.051(-0.227, 0.330)	0.722	20	-0.145(-0.479, 0.189)	0.407	21	-0.04(-0.492, 0.411)	0.863	18	0.036(-0.313, 0.386)	0.841
	Weighted median	20	0.063(-0.028, 0.153)	0.174	20	-0.04(-0.150, 0.069)	0.47	21	0.109(-0.018, 0.236)	0.092	18	-0.01(-0.133, 0.114)	0.875
	Maximum likelihood	20	0.085(0.020, 0.149)	**0.01**	20	-0.049(-0.124, 0.027)	0.205	21	0.101(0.015, 0.186)	**0.022**	18	-0.035(-0.118, 0.049)	0.416

IVW, inverse variance–weighted method; fe, fixed-effects model; re, multiplicative random-effects model; BMI, body mass index; WHR, waist-hip ratio; TIBC, total iron-binding capacity; TSAT, transferrin saturation; SNP, Single-nucleotide polymorphism. The bold font means P < adjusted p-values (0.013).

**Figure 4:**
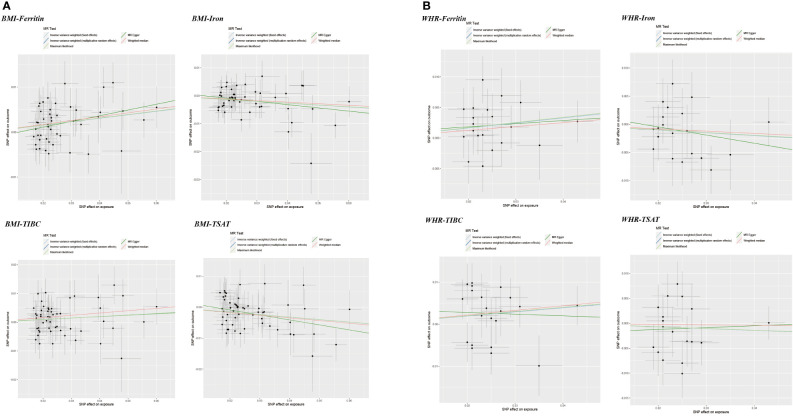
Scatter plots of Mendelian randomization analyses of the association between genetically predicted obesity-related traits and iron status. **(A)** BMI - serum ferritin, serum iron, TIBC and TSAT; **(B)** WHR - serum ferritin, serum iron, TIBC and TSAT.

**Figure 5:**
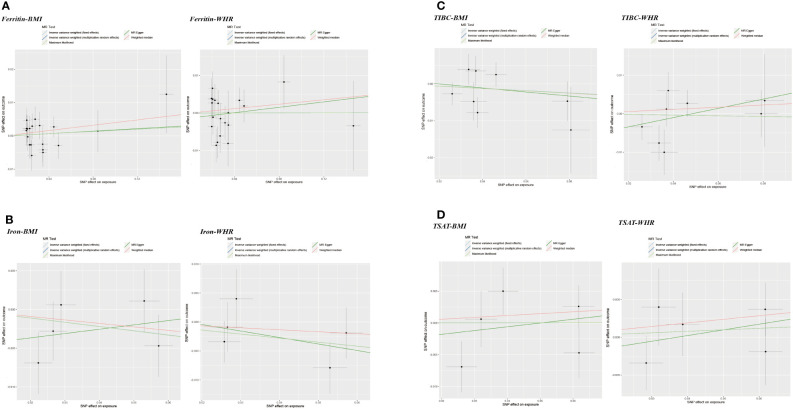
Scatter plots of Mendelian randomization analyses of the association between genetically predicted iron status and obesity-related traits. **(A)** serum ferritin – BMI and WHR; **(B)** serum iron – BMI and WHR; **(C)** TIBC – BMI and WHR; **(D)** TSAT - BMI and WHR.

In addition, WHR was associated with the serum ferritin levels (β = 0.082, 95%CI: 0.023, 0.141, P = 0.006). However, MR Egger and weighted median did not provide consistent results (**Table 2** and **Supplemental Tables 13**). The results of the MR Steiger directionality test illustrated the accuracy of the causal direction of the current MR analysis. The forest plots and scatter plots of MR analysis were used to present the data (**Figures 2**–**5**).

## 4 Discussion

In this two-sample bidirectional MR study, we used the summary GWAS data from the European population to investigate the causal association between iron status and obesity-related traits. Our findings showed that genetically predicted iron status biomarkers were not associated with a higher risk of obesity. For the reverse analysis, genetically predicted BMI was associated with decreased serum iron and TSAT levels and increased serum ferritin levels. Moreover, the inference of causality between WHR and serum ferritin levels cannot be confirmed using additional methods, suggesting that the evidence is insufficient and conclusion should be drawn carefully. To the best of our knowledge, this is the first study on causality between iron status and obesity-related traits using MR approach. The stability of our results lies in the following: We identified and removed the outliers using multiple methods and obtained a stable positive result. In addition, the same results can be obtained using various MR methods, which can increase the stability of the results.

A previous study indicated that iron content in the adipose tissue of obese patients appears to be increased (36). A cross-sectional study showed a high burden of obesity in women of reproductive age with iron deficiency anemia (IDA) (37). In addition, animal studies revealed that dietary iron deficiency could modulate diet-induced weight gain in mice (38). However, numerous other factors contribute to obesity, including total energy intake. Thus, the previous observational studies might influence by unmeasured confounders. Our findings suggest no causal association between iron status biomarkers and the risk of obesity. Further investigation is still needed into the effect of iron status on obesity and the potential mechanism.

We found evidence that BMI was associated with decreased serum iron and TSAT levels and increased serum ferritin levels. Our results are in line with previous clinical studies that the serum iron reserves in obese participants are consistently higher, and the decrease in BMI improves the iron state and absorption by reducing hepcidin levels (39, 40). In addition, epidemiological studies showed that BMI was negatively associated with iron, TIBC, and TSAT levels and positively associated with serum ferritin levels (41, 42). Potential mechanism might be proposed to explain this association. The association between BMI and iron status could be attributed to inflammation and hepcidin. In the set of ongoing inflammation, the serum ferritin levels increase while the serum iron levels decrease, which impacts iron homeostasis (43). Some researchers suggest that hepcidin may involve in mediating the pathways between inflammation and iron status (44). Hepcidin is regarded as a central regulatory peptide of intestinal iron absorption and iron recycling and is regulated by interleukin-6 (IL-6) secreted by adipose tissues (45). Therefore, we speculated that obesity might reduce iron absorption and storage by mediating inflammation and decreasing hepcidin levels. In addition, Failla et al. observed lower concentrations of iron in obese mice, which may be due to an adaptive response to expanded blood volume (46). Our results provide a theoretical basis for further research on lowering BMI to the prevention of abnormal iron metabolism. Further studies should provide insights into the pathogenesis of BMI in ID.

In addition, the causal estimate of WHR and serum ferritin did not yield stable results in our analysis. Studies on the impact of WHR on the iron status are still limited. Waist circumference, as the main indicator of concentric obesity, is suggested to be positively but insignificantly associated with plasma iron, TSAT, and ferritin concentrations (47). The potential mechanism may be that the massive release of leptin from the adipose tissue causes increased hepcidin release, resulting in iron depletion or low circulating iron concentrations (47). Nevertheless, more studies are needed to confirm this result.

The main strength of our study is the use of inborn genetic instruments to proxy the exposure trait, which could be less influenced by confounding or reverse causation. However, our study has several limitations. First, the data of genetic variants relied primarily on the GWAS of iron status and obesity-related traits from European descent, which may limit our finding from being fully representative of the whole population. However, restricting participants’ descent can minimize the risk of confounding by population admixture. Second, the use of summary-level data limits the range of analyses that can be performed, including the nonlinear relationship between exposure and outcomes and the stratified analysis of either age or sex. Last, we are not able to estimate the degree of overlap of participants between the datasets in the current study. However, the genetic instrument used in the current study was judged by F-statistics, with a strong instrument defined as an F > 10, which could minimize the bias from sample overlap (48).

## 5 Conclusion

In summary, we found that the genetically predicted BMI may contribute to the decreased serum ferritin levels and increased serum iron and TSAT levels. However, there was no evidence to support the causality of iron status on the risk of obesity. This may help inform clinicians that early intervention to improve iron status should involve appropriate weight control, which may be an effective measure and potential strategy.

## Data availability statement

The original contributions presented in the study are included in the article/**Supplementary Material**. Further inquiries can be directed to the corresponding author.

## Author contributions

ZZ, HZ conceived and designed the study. ZZ, HZ, KC, and CL performed experiments and wrote the manuscript. ZZ interpreted the data and prepared the manuscript. All authors read and approved the final manuscript.
