# Supplementary material for: Iron status and obesity-related traits: A two-sample bidirectional Mendelian randomization study
Source: Front Endocrinol (Lausanne). 2023 Feb 14;14:985338. doi: 10.3389/fendo.2023.985338 (PMC9971727; doi:10.3389/fendo.2023.985338)
Supplement: Supplementary file 2 [file Image_1.pdf]

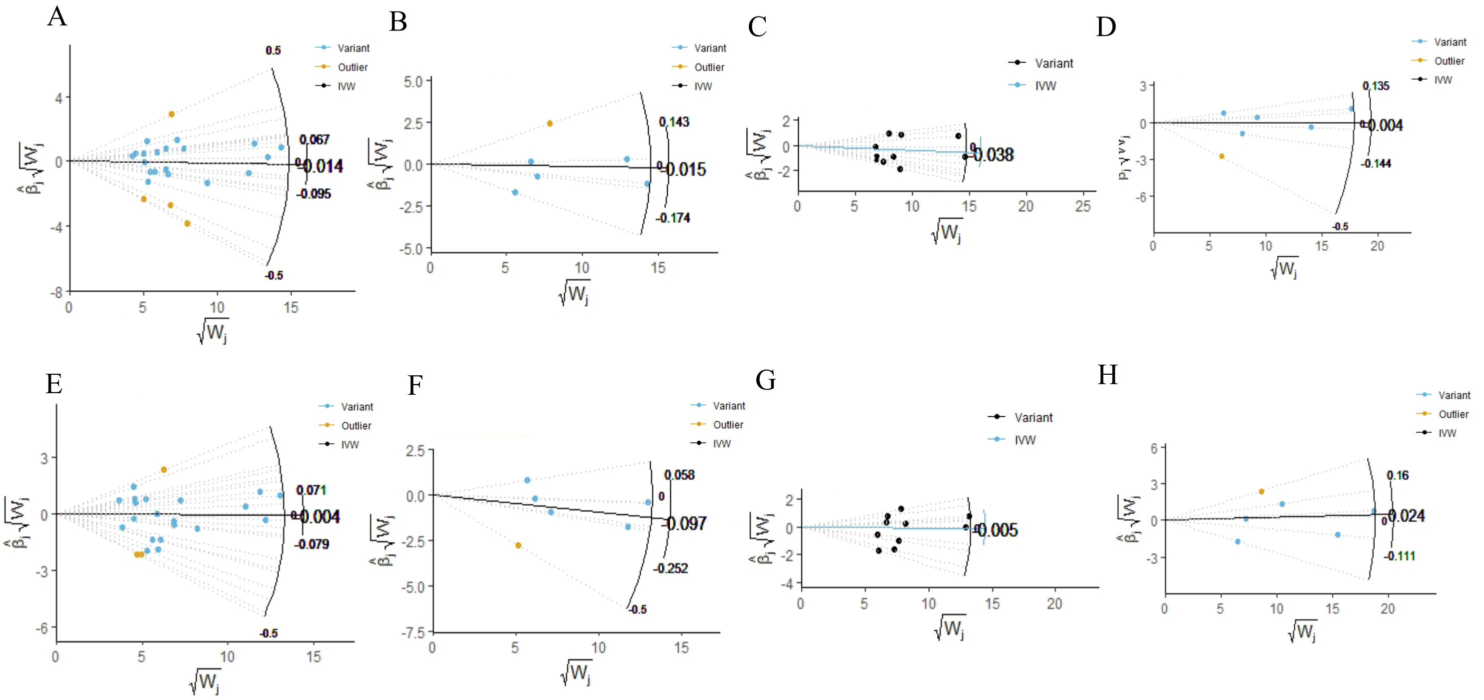

Supplemental Figure 1 Result for the RadialMR analysis in the SNPs (RadialMR plot). Association of genetically instrumented A) serum Ferritin; B) serum iron; C) TIBC; D) TSAT (exposure) with BMI (outcome) and E) serum Ferritin; F) serum iron; G) TIBC; H) TSAT (exposure) with WHR (outcome). Abbreviations: IVW, inverse variance weighting.

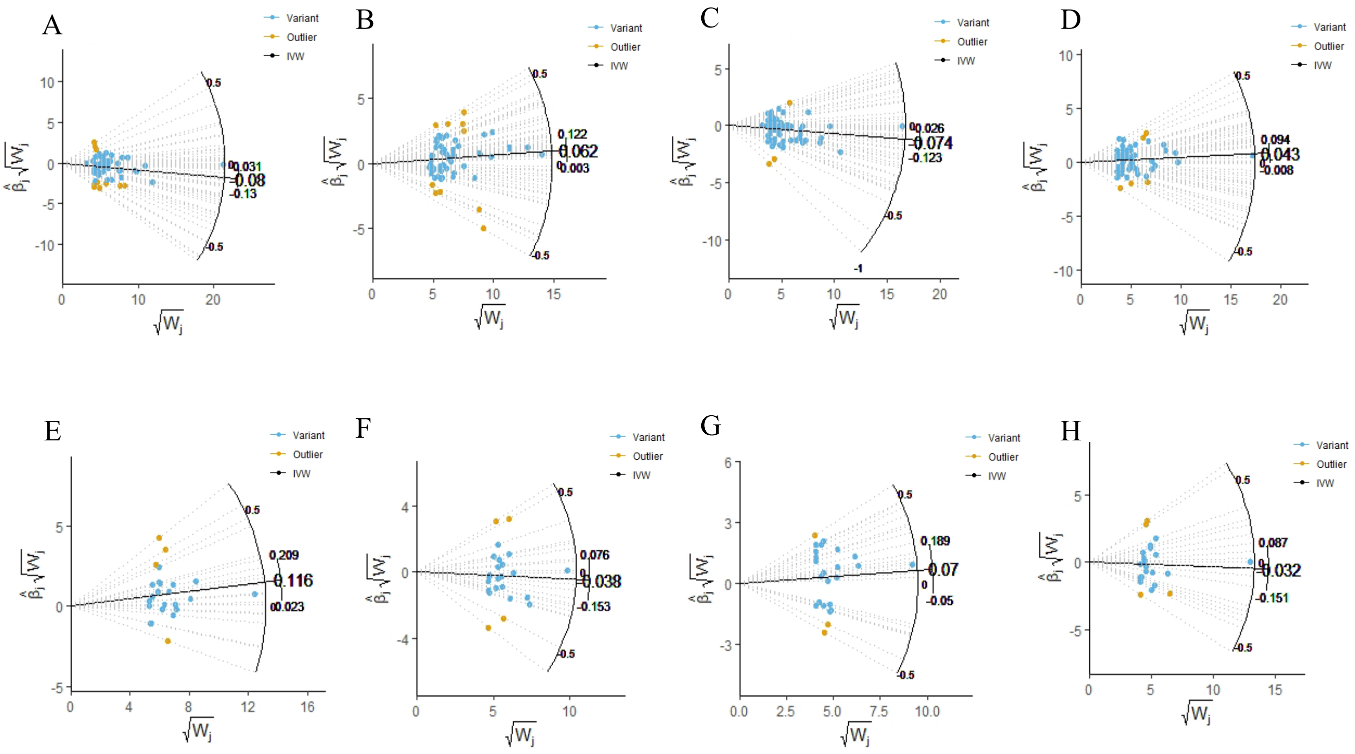

Supplemental Figure 2 Result for the RadialMR analysis in the SNPs (RadialMR plot). Association of genetically instrumented BMI (exposure) with A) serum Ferritin; B) serum iron; C) TIBC; D) TSAT (outcome) and WHR (exposure) with E) serum Ferritin; F) serum iron; G) TIBC; H) TSAT (outcome). Abbreviations: IVW, inverse variance weighting.

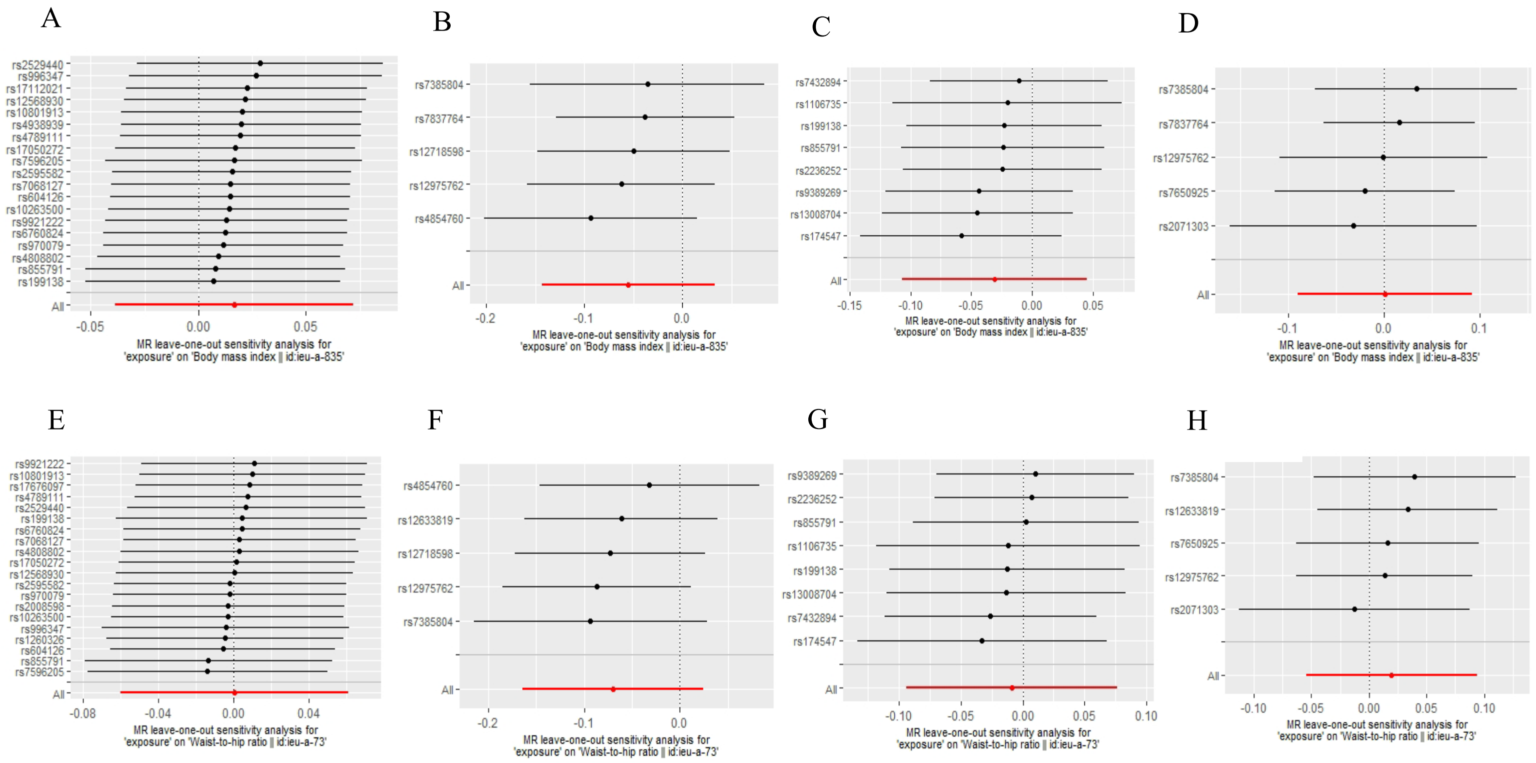

Supplemental Figure 3 Result for leave-one-out analysis of the forward causal effect (Forest plots). Association of genetically instrumented A) serum Ferritin; B) serum iron; C) TIBC; D) TSAT (exposure) with BMI (outcome) and E) serum Ferritin; F) serum iron; G) TIBC; H) TSAT (exposure) with WHR (outcome).

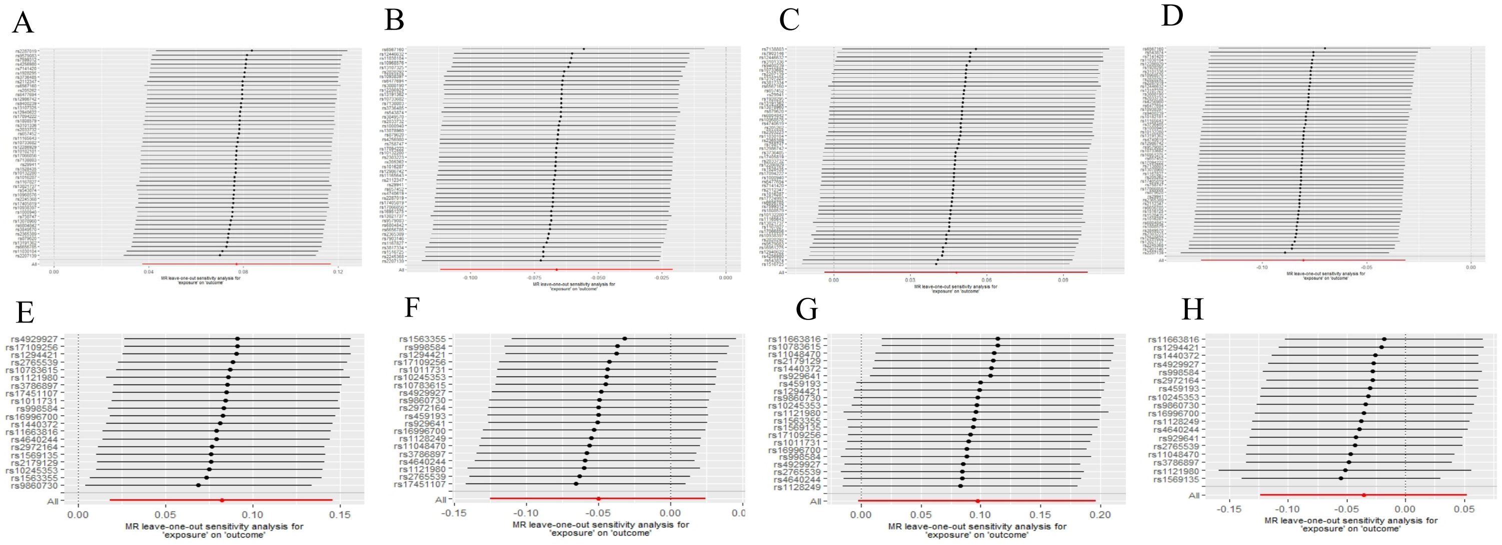

Supplemental Figure 4 Result for leave-one-out analysis of the reverse causal effect (Forest plots). Association of genetically instrumented BMI (exposure) with A) serum Ferritin; B) serum iron; C) TIBC; D) TSAT (outcome) and WHR (exposure) with E) serum Ferritin; F) serum iron; G) TIBC; H) TSAT (outcome).
